# Promoting reactive oxygen species accumulation to overcome tyrosine kinase inhibitor resistance in cancer

**DOI:** 10.1186/s12935-024-03418-x

**Published:** 2024-07-09

**Authors:** Wei Lin, Xiaojun Wang, Mingxin Diao, Yangwei Wang, Rong Zhao, Jiaping Chen, Yongde Liao, Qinghong Long, Yunchong Meng

**Affiliations:** 1 Department of Thoracic Surgery, Union Hospital, Tongji Medical College, Huazhong University of Science and Technology, Jiefang Avenue, Jianghan District, Wuhan, Hubei 430022 P.R. China; 2 Department of Thoracic Surgery, Fujian Medical University Union Hospital, Fuzhou, China; 3 Department of Cardiothoracic Surgery, Third Affiliated Hospital of Kunming Medical University (Yunnan Cancer Hospital), Kunming, Yunnan China; 4 Department of Internal Medicine, Renmin Hospital, Wuhan University, Wuhan, 430022 China

**Keywords:** Cancer, Tyrosine kinase inhibitor, Drug resistance, Reactive oxygen species, ROS homeostasis, Antioxidant pathway

## Abstract

**Background:**

In tumor treatment, protein tyrosine kinase inhibitors (TKIs) have been extensively utilized. However, the efficacy of TKI is significantly compromised by drug resistance. Consequently, finding an effective solution to overcome TKI resistance becomes crucial. Reactive oxygen species (ROS) are a group of highly active molecules that play important roles in targeted cancer therapy including TKI targeted therapy. In this review, we concentrate on the ROS-associated mechanisms of TKI lethality in tumors and strategies for regulating ROS to reverse TKI resistance in cancer.

**Main body:**

Elevated ROS levels often manifest during TKI therapy in cancers, potentially causing organelle damage and cell death, which are critical to the success of TKIs in eradicating cancer cells. However, it is noteworthy that cancer cells might initiate resistance pathways to shield themselves from ROS-induced damage, leading to TKI resistance. Addressing this challenge involves blocking these resistance pathways, for instance, the NRF2-KEAP1 axis and protective autophagy, to promote ROS accumulation in cells, thereby resensitizing drug-resistant cancer cells to TKIs. Additional effective approaches inducing ROS generation within drug-resistant cells and providing exogenous ROS stimulation.

**Conclusion:**

ROS play pivotal roles in the eradication of tumor cells by TKI. Harnessing the accumulation of ROS to overcome TKI resistance is an effective and widely applicable approach.

**Graphical Abstract:**

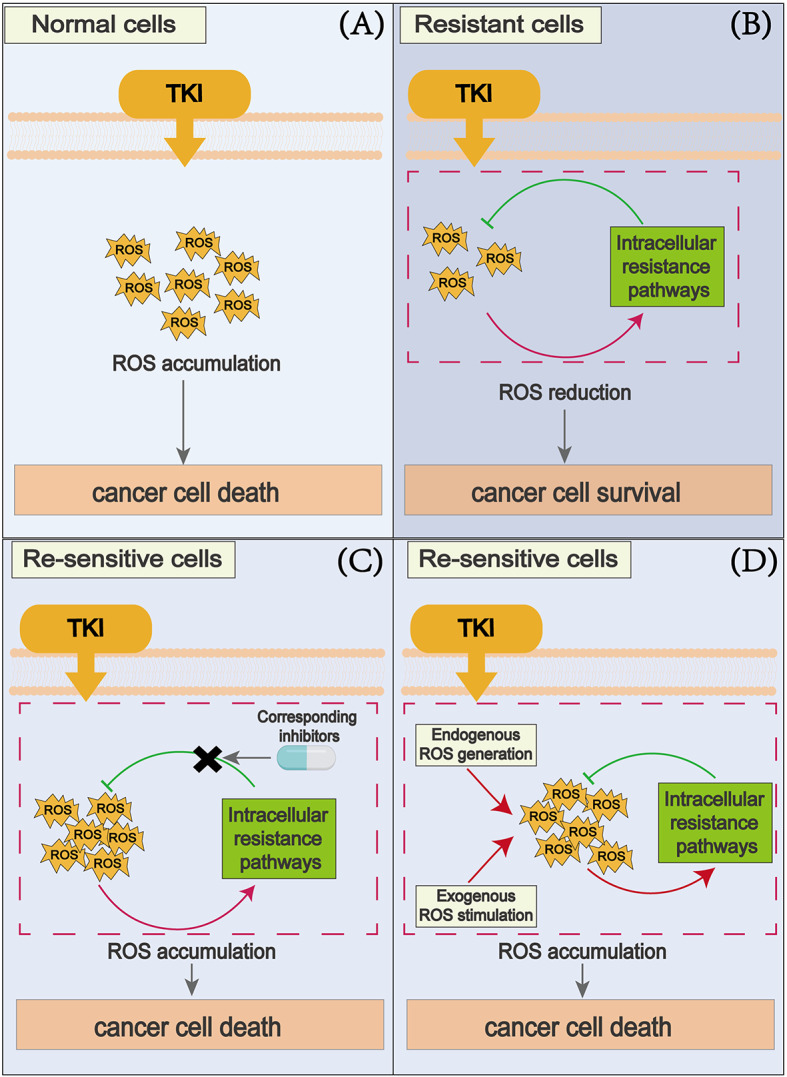

## Background

Protein tyrosine kinases are protein kinases that phosphorylate tyrosine residues on downstream proteins in signal transduction, playing a crucial role in cellular life activities [1]. Protein tyrosine kinases are categorized into receptor tyrosine kinases and nonreceptor tyrosine kinases, both of which are implicated in tumor-related activities such as growth, proliferation, metastasis, and angiogenesis [2, 3]. Among receptor tyrosine kinases are the epidermal growth factor receptor (EGFR), vascular endothelial growth factor receptor (VEGFR), and fibroblast growth factor receptor (FGFR); and in the nonreceptor tyrosine kinases group are anaplastic lymphoma kinase (ALK), ABL kinase, and Src kinase. Numerous targeted inhibitors for these kinases have been developed and are widely used in clinical cancer therapies with good therapeutic effects. Nonetheless, the development of drug resistance limits the efficacy of TKIs. Resistance to TKIs often results from different gene mutations in the targets or the activation of alternative pathways, and these complex mechanisms make it difficult to develop widely effective means to overcome resistance [4, 5]. Consequently, it is necessary to summarize the commonalities of TKI resistance in cancer.

ROS are a group of highly active molecules containing oxygen, such as singlet oxygen (1O_2_), superoxide (O_2_^•-^), hydroxyl radical (OH^•^) and hydrogen peroxide (H_2_O_2_). Endogenous ROS arise from various sources such as mitochondrial metabolism, peroxisomes, and the function of transmembrane NADPH oxidases (NOXs) [6]. ROS play multiple roles in tumor cells. Low ROS levels generally support tumor initiation, progression, and survival, whereas elevated ROS levels tend to cause oxidative harm on DNA, proteins, and lipids, often resulting in cell death [7, 8]. The induction of high ROS levels in cancer cells, aimed at triggering regulated cell death, represents one of the main effects of radiotherapy and chemotherapy [9]. Similarly, in targeted cancer therapies, ROS-mediated tumor cell death is also universal and closely linked to the development of drug resistance, such as ROS and EGFR [10, 11]. Consequently, as an important factor in tumor treatment response, summarizing the mechanisms of ROS in the TKI treatment process and drug resistance may be a breakthrough in overcoming widespread TKI resistance See Table 1.

**Table 1:** FDA approved tyrosine kinase inhibitors therapeutics for cancers

TKI	Generation	Primary targets	Cancer	Year approved
Herceptin	First	HER2	breast cancer, gastric cancer	1998
Imatinib	First	BCR-ABL, PDGFR, SCFR, c-kit	CML, gastrointestinal stromal tumor	2001
Gefitinib	First	EGFR	NSCLC	2003
Erlotinib	First	EGFR (exon 19 deletions, exon 21 L858R mutations)	NSCLC, pancreatic cancer	2004
Sorafenib	First	Raf, VEGFR, PDGFR, c-kit, FLT-3	RCC, HCC, differentiated thyroid carcinoma	2005
Vemurafenib	First	RAF	melanoma	2011
Midostaurin	First	FLT-3	AML	2017
Larotrectinib	First	TRKA/B/C	solid tumors with NTRK fusion proteins	2018
Erdafitinib	First	FGFR	urothelial carcinoma	2019
Sunitinib	Second	VEGFR, PDGFR, c-kit, FLT-3	GIST, RCC, pancreatic neuroendocrine tumors	2006
Dasatinib	Second	BCR-ABL, Src, c-kit, PDGFR	CML	2006
Lapatinib	Second	EGFR, HER2	breast cancer	2007
Nilotinib	Second	BCR-ABL, PDGFR, c-kit	CML	2007
Pazopanib	Second	VEGFR, PDGFR, FGFR, c-kit	RCC, soft tissue sarcoma	2009
Crizotinib	Second	ALK, ROS1	NSCLC, anaplastic large cell lymphoma	2011
Vandetanib	Second	VEGFR, EGFR	medullary thyroid cancer	2011
Axitinib	Second	VEGFR	RCC	2012
Afatinib	Second	EGFR, HER2	NSCLC	2013
Ceritinib	Second	ALK	NSCLC	2014
Alectinib	Second	ALK, RET	NSCLC	2015
Neratinib	Second	EGFR, HER2	breast cancer	2017
Dacomitinib	Second	EGFR, HER1, HER2, HER4	NSCLC	2018
Gilteritinib	Second	FLT-3	AML	2018
Entrectinib	Second	TRKA/B/C, ALK, ROS1	solid tumors with NTRK fusion proteins, NSCLC	2019
Pexidartinib	Second	FLT-3, CSF1R, c-kit	tenosynovial giant cell tumor	2019
Capmatinib	Second	MET	NSCLC	2020
Infigratinib	Second	FGFR	cholangiocarcinoma	2021
Futibatinib	Second	FGFR	intrahepatic cholangiocarcinoma	2022
Regorafenib	Third	Raf, RET, PDGFR, FGFR	CRC, gastrointestinal stromal tumor, HCC	2012
Osimertinib	Third	EGFR (T790M mutations, exon 19 deletions, exon 21 L858R mutations)	NSCLC	2015
Lenvatinib	Third	VEGFR, FGFR, PDGFR	differentiated thyroid carcinoma, RCC, HCC, endometrial carcinoma	2015
Brigatinib	Third	ALK, ROS1	NSCLC	2017
Lorlatinib	Third	ALK, ROS1	NSCLC	2018
Tepotinib	Third	MET	NSCLC	2021
Tivozanib	Third	VEGFR, PDGFR	RCC	2021
Asciminib	Third	BCR-ABL	CML	2021
Mobocertinib	Third	EGFR (exon 20 insertion mutations)	NSCLC	2021
Pirobrutinib	Third	BTK	mantle cell lymphoma	2023

## The ROS-mediated mechanisms of cancer cell killing by different TKIs

**Fig. 1:**
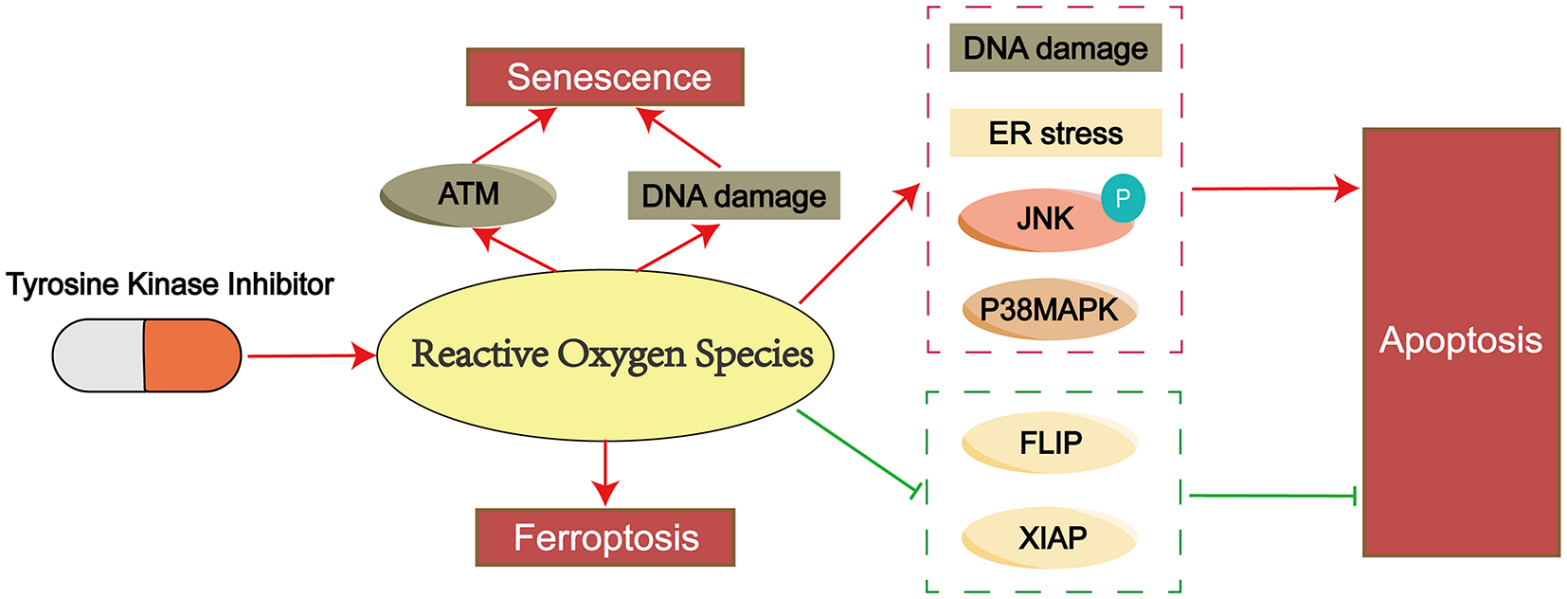
Treatment with TKIs leads to the accumulation of ROS in cancer cells through various mechanisms. High levels of ROS induce apoptosis through the activation of various pathways, including JNK and P38MAPK, endoplasmic reticulum stress, DNA damage, and repression of antiapoptotic proteins XIAP and FLIP. Additionally, the increase of ROS induces ferroptosis in cancer cells. Furthermore, ROS activates ATM and cause DNA damage, leading to cellular senescence and death

ROS play many important roles in cells. Accumulating ROS can induce a cascade of cellular events, especially mitochondrial damage and apoptosis [12]. This phenomenon also frequently occurs during TKI treatment. In this section, we aim to illustrate the tumor cell killing effects of different types of TKIs through ROS-related mechanisms (Fig. 1).

### ErbB inhibitors

Commercial ErbB inhibitors mainly target two targets: ErbB1 (EGFR) and ErbB2 (HER2).

Gefitinib, as the first marketed EGFR-TKI, has achieved good clinical efficacy against EGFR 19 exon deletion (19Del) and L858R mutation in exon 21 (L858R). Under gefitinib treatment, lung cancer cells show a rise in ROS levels over time, leading to mitochondrial dysfunction [13]. In glioma, gefitinib promotes ROS production via NOX2 and NOX4, triggering endoplasmic reticulum stress and activating the apoptosis signal-regulating kinase 1/c-Jun N-terminal kinase/Noxa (Ask1/JNK/Noxa) pathway, which results in caspase-dependent apoptosis [14]. Erlotinib, another selective EGFR-TKI, increase the ROS levels in lung cancer cells, promoting JNK phosphorylation that activates c-Jun and caspase-3 to lead to apoptosis [15]. In head and neck cancer (HNSCC), erlotinib elevates NOX4 expression and hydrogen peroxide production, which not only cause cytotoxicity but also protective autophagy [16, 17]. For serine/threonine kinase 11-deficient non-small cell lung cancer (NSCLC) cells, erlotinib suppresses growth and induces apoptosis through the activation of adenosine monophosphate-activated protein kinase (AMPK) and suppression of the mammalian target of rapamycin (mTOR) signaling, due to increased ROS and associated mitochondrial damage [18]. Furthermore, the third-generation EGFR-TKI, Osimertinib, has been shown to elevate the ROS levels in NSCLC cells, leading to mitochondrial impairment and cellular apoptosis [19].

Herceptin (trastuzumab) is a humanized monoclonal antibody targeting the extracellular domain of HER2 [20]. In lung cancer cells, herceptin is known to promote ROS production, which activates caspase 3/7 and leads to apoptosis [21]. However, in breast cancer, it inhibits survivin via the HER2/β-catenin/T-cell factor 4-survivin pathway, resulting in apoptosis [22]. HER2 is able to inhibit protein kinase B (PKB or AKT)- and protein kinase C α-dependent pathways [23] or promote GPX1 expression to initiate ROS clearance [24], but the blockade of HER2 by herceptin leads to intracellular accumulation of ROS and cell death, which is an important reason for cardiac toxicity during herceptin treatment.

Afatinib, lapatinib and neratinib are all pan-ErbB inhibitors, that can block both the EGFR and HRE2 pathways [25–27]. Afatinib acts on EGFR and activates the P38MAPK signaling pathway to inhibit xCT, causing ROS increase, lipid peroxidation, and cell ferroptosis in gastric cancer cells [28]. Likewise, afatinib promotes accumulation of intracellular ROS, followed by apoptosis in lung cancer cells [29]. In hepatoma cells, lapatinib treatment results in mitochondrial toxicity, evidenced by raised levels of mitochondrial O2- and cytoplasmic H2O2 [30]. Similarly, neratinib induces ferroptosis in acute myeloid leukemia (AML) cells, which is characterized by increased ROS and malondialdehyde content, enhanced Fe^2+^ activity, and downregulated GPX4 and ferritin heavy chain 1 expression [31].

### ALK inhibitors

Crizotinib is a highly potent and selective ALK/c-MET dual inhibitor [32]. Crizotinib treatment leads to the accumulation of intracellular ROS, causing further mitochondrial depolarization and activating the ROS-dependent apoptotic pathway, thereby killing cervical cancer cells [33]. In another study involving human alveolar rhabdomyosarcoma cells, crizotinib displayed the potential to induce apoptosis in a dose-dependent manner through ROS accumulation, as indicated by caspase 3 activation and PARP proteolytic cleavage downregulation [34].

### VEGFR inhibitors

Axitinib is a second-generation VEGFR inhibitor approved by the US Food and Drug Administration in 2012 for the treatment of patients with advanced renal cell carcinoma (RCC) [35, 36]. Axitinib significantly inhibits the activity of RCC by promoting the release of ROS and inducing cancer cell apoptosis [37]. Moreover, the accumulation of ROS induced by axitinib causes a DNA damage response and oxidative stress-dependent activation of the ataxia telangiectasia mutated (ATM) kinase, triggering cellular senescence [38, 39]. Pazopanib is a new multitargeted receptor tyrosine kinase inhibitor that targets VEGFR1, VEGFR2, VEGFR3, and the platelet-derived growth factor receptor (PDGFR) [40]. Pazopanib induces small cell lung cancer cell apoptosis through the endoplasmic reticulum stress (ER stress) process via upregulation of ROS levels [41]. Regorafenib is also a broad-spectrum tyrosine kinase inhibitor targeting VEGFR1-3, TEK receptor tyrosine kinase (TIE-2), etc. Regorafenib increases ROS generation by promoting NOX5 expression and activates ROS-mediated ER stress, c-Jun and P38MAPK signaling pathways [42]. In addition, regorafenib treatment promotes Bim-mediated ROS accumulation and cancer cell apoptosis via blocking AKT-mediated FOXO3a nuclear export [43].

### BCR-ABL inhibitors

Imatinib is the first tyrosine kinase inhibitor to be marketed and used to treat chronic myeloid leukemia (CML) by targeting the oncogenic protein BCR-ABL. In CML, the BCR-ABL gene induces the production of ROS, cause DNA damage and regulate the DNA repair process, which leads to genomic instability, increased gene mutations, and tumor progression [44]. For leukemia with BCR-ABL mutations, imatinib directly inhibits the BCR-ABL protein to reduce ROS-related processes. However, imatinib also has an opposing impact on ROS. It increases ROS and induces ER stress by inhibiting PDGFR phosphorylation, which activates JNK phosphorylation, leading to mitochondrial-related cell apoptosis in gastric cancer cells [45]. Imatinib elevates intracellular peroxide levels and activates JNK and p38 protein phosphorylation, enhancing caspase 3/9 enzyme activity and disrupting mitochondrial membrane potential, which drives ROS-dependent apoptosis in melanoma B16F0 cells [46]. Nilotinib, a second-generation TKI targeting imatinib-resistance CML patients, increases the activity of glycogen synthase kinase 3β (GSK3β) by phosphorylating the Ser473 site of AKT, resulting in NOX4 upregulated, ROS accumulation and cell apoptosis [47]. In addition, it is interesting to note that H_2_O_2_ downregulates the levels of the antiapoptotic proteins FLICE-like inhibitory protein (FLIP) and X-linked inhibitor of apoptosis protein (XIAP) in imatinib-resistant K562 cells, which tends to promote CML cell apoptosis [48]. These findings illustrate the dual nature of ROS in both promoting tumor growth and contributing to tumor cell death.

### FGFR inhibitors

Lenvatinib is an oral multitarget receptor tyrosine kinase inhibitor that is approved for the treatment of hepatocellular carcinoma (HCC) and metastatic renal cell carcinoma [49, 50]. Lenvatinib inhibits the expression of xCT and GPX4 by inhibiting FGFR4, leading to the accumulation of lipid ROS and ultimately ferroptosis in HCC [51]. Another study found that lenvatinib prevents nuclear translocation of β-catenin to inhibit the expression of GPX2, thereby increasing the levels of ROS in HCC cells and furthering cancer cell apoptosis [52]. Erdafitinib, a novel FGFR inhibitor, disturbed lysosome functions by altering the matrix pH value, which resulted in blocked autolysosome degradation and autophagy. Blocked autophagy elevated intracellular ROS levels, causing DNA damage accumulation and apoptosis, which account for the cytotoxicity of erdafitinib in FGFR3- altered bladder cancer [53].

### Contrary view

In most studies, TKIs cause tumor cell death by causing intracellular ROS accumulation, but there are still some studies that suggest that ROS plays a role in promoting tumor progression. For instance, treating patients with wide-type EGFR lung cancer with cisplatin revealed that TKIs produced neither a synergistic nor an enhancing effect on platinum-based chemotherapy, with some evidence pointing to a possible antagonistic effect [54]. Subsequent research indicated that gefitinib suppressed the EGFR-ERK/AKT signaling pathway, which activated FOXO3a and lowered ROS levels, thereby obstructing the caspase-independent cell death prompted by cisplatin [55]. Pedunculoside, a triterpene saponin extracted from *Ilex rotunda* Thunb, downregulates epithelial-mesenchymal transition (EMT)-related protein expression through the MAPK and NRF2 pathways, decreases ROS production, and counteracts NSCLC metastasis [56]. Moreover, ROS have been reported to induce tumor growth by upregulating DNMT1 expression and downregulating miR-199a and miR-125b expression, thereby promoting the expression of ErbB2 and ErbB3 [57]. The promotion of tumor progression by ROS is more evident in BCR-ABL positive cells. As mentioned above, ROS induced by BCR-ABL causes chronic oxidative DNA damage and stimulates homologous recombination repair, leading to a high gene mutation rate, which is also one of the important reasons for the emergence of imatinib resistance in leukemia [58, 59].

In conclusion, TKIs elevate ROS levels within tumor cells through various mechanisms, as detailed in Table 2, leading to apoptosis, ferroptosis, and increased cytotoxicity. However, in certain instances, the production of ROS can also mediate genomic instability, metastasis, and the inhibition of apoptosis in cancer cells. Therefore, understanding the specific role of ROS requires considering many factors, such as the type, level, location, and persistence of ROS, as well as the origin, environment, and stage of the tumor [6].

**Table 2:** Summary of TKI-induced ROS accumulation in various cancer model systems

TKIs	Cancers	Mechanism to enhance ROS accumulation	Model systems	References
Gefitinib	Lung cancer	Unspecified	H1650 cell line	[13]
Gefitinib	Glioma	Upregulating NOX2 and NOX4 to promote ROS generation	U87 and H4 cell lines	[14]
Erlotinib	NSCLC	Unspecified	A549 cell line	[15]
Erlotinib	HNSCC	Upregulating NOX4 to promote ROS generation	FaDu, Cal-27 and SQ20B cell lines; mouse xenograft model	[16]
Osimertinib	NSCLC	Unspecified	HCC827 and H1975 cell lines; mouse xenograft model	[19]
Herceptin	NSCLC	Unspecified	Calu-3 cell line	[21]
Afatinib	Gastric cancer	Activating the P38MAPK signaling pathway to inhibit xCT, then causing ROS increase	AGS and BGC-823 cell lines	[28]
Afatinib	Lung cancer	Unspecified	H1650 and H1975 cell lines	[29]
Lapatinib	Liver cancer	Unspecified	HepG2 cell line	[30]
Neratinib	Acute myeloid leukemia	Unspecified	HL-60 cell line	[31]
Crizotinib	Cervical cancer	Unspecified	HeLa and SiHa cell lines	[33]
Crizotinib	Rhabdomyosarcoma	Unspecified	RH4 and RH30 cell lines	[34]
Axitinib	Renal cell carcinoma	Unspecified	ACHN, A-498 and Caki-2 cell lines	[37, 38]
Pazopanib	Small cell lung cancer	Unspecified	NCI-H446 and NCI-H82 cell lines; mouse xenograft model	[41]
Regorafenib	NSCLC	Upregulating NOX5 to promote ROS generation	H1299 and PC-9 cell lines; mouse xenograft model	[42]
Regorafenib	Various cancer	Promoting FOXO3a nuclear localization to upregulate Bim expression, then causing ROS increase	MCF-7 cell line	[43]
Imatinib	Gastric cancer	Inhibiting PDGFR phosphorylation and then causing ROS increase	AGS cell line	[45]
Lenvatinib	Hepatocellular carcinoma	Inhibiting FGFR4 to suppress xCT, then causing ROS increase	HuH7 and Hep3B cell lines	[51]
Lenvatinib	Hepatocellular carcinoma	Preventing nuclear translocation of β-catenin to inhibit GPX2 expression	HepG2 and HuH7 cell lines; mouse xenograft model	[52]
Erdafitinib	Bladder cancer	Blocking autolysosome degradation and then causing ROS increase	RT-112 cell line; mouse xenograft model	[53]

## Promoting ROS accumulation to overcome TKI resistance

**Fig. 2:**
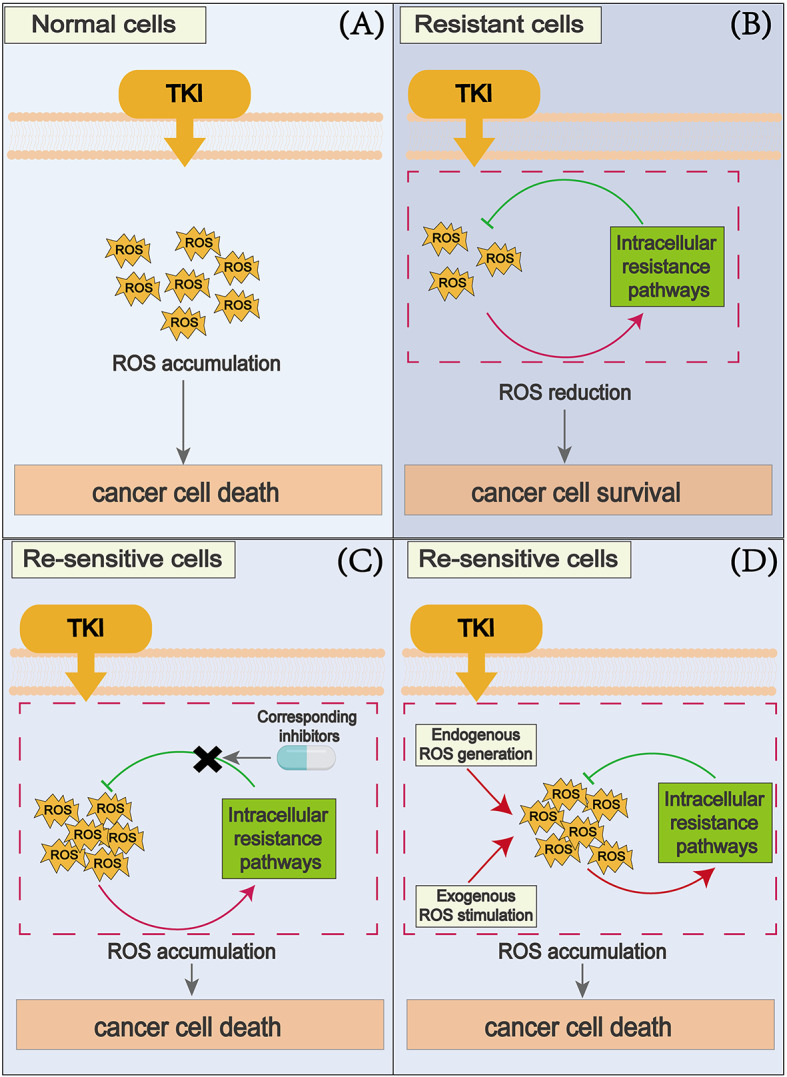
Schematic representation showing strategies to promote ROS accumulation and overcome TKI resistance. (**A**) In normal cancer cells, TKI treatment leads to the accumulation of intracellular ROS and results in cell death. (**B**) In TKI-resistant cancer cells, ROS induce the activation of intracellular resistance pathways. These activated pathways feedback to inhibit the accumulation of ROS, leading to a decrease in ROS levels, thereby allowing cells to become resistant to oxidative damage and survive. (**C**) By blocking resistance pathways with corresponding inhibitors, the accumulation of ROS induced by TKIs can be restored, making cancer cells re-sensitized to TKIs. (**D**) Promoting endogenous ROS generation or providing exogenous ROS stimuli can re-induce the accumulation of ROS in cancer cells, making them re-sensitized to TKIs

When the concentration of ROS increases beyond the physiological concentration, they may damage cells. To protect themselves from ROS damage, cells initiate appropriate countermeasures to cope with ROS elevation and maintain cellular oxidative stress homeostasis [60]. Under TKI stress, cancer cells trigger anti-ROS pathways to cope with the accumulation of ROS, and the activation of these antioxidant pathways contribute to the development of drug resistance in cancer cells. Therefore, blocking these antioxidant pathways in cancer cells is an effective strategy to overcome TKI resistance. Additionally, activating other intracellular ROS generation pathways or providing exogenous ROS stimulation is also a choice for synergistic TKIs to exert killing effects (Fig. 2). Here we summarize some mechanisms for overcoming TKI resistance by promoting intracellular ROS accumulation.

### Blocking intracellular resistance pathways to overcome TKI resistance

#### Autophagy

Autophagy is a catabolic degradation process of cells under internal or external stress and has been proposed as a cell death mechanism, called programmed cell death type II [61, 62]. Autophagy is the process of transferring cytoplasmic macromolecules, aggregated proteins, damaged organelles or pathogens to lysosomes, which are then digested and decomposed into nucleotides, amino acids, fatty acids, sugars and ATP, and finally recycling [63–65]. The role of autophagy in cancer is complex, including tumor occurrence, development, and maintenance of malignancy [63]. Oxidative stress is a major trigger of autophagy, with ROS participating in signal transduction in autophagic cells [66]. Autophagy plays different roles in TKI treatment, including toxic autophagy-mediated cell death and protective autophagy-mediated TKI resistance.

Autophagy has been frequently observed in tumor treatment with TKIs, such as afatinib for head and neck cancer [67], osimertinib for lung cancer [68], lenvatinib for liver cancer [69], and erlotinib for head and neck cancer [17]. Autophagy-dependent cell death is usually accompanied by an increase in autophagy markers and the accumulation of autophagosomes [70]. Lapatinib, in combination with the BCL-2 family inhibitor obatoclax (GX15-070), kills resistant breast cancer cells through mTOR inhibition and P38MAPK activation, resulting in ROS production, ER stress signal activation, and stimulation of toxic autophagy [71, 72]. When lenvatinib is administered alongside the histone deacetylase (HDAC) inhibitor entinostat, it activates ATM and the elongation initiation factor 2α (eIF2α) via ROS. This activation increases Beclin1 and autophagy-related 5 expression, resulting in the enhanced formation of toxic autophagosomes and reduced expression of protective mitochondrial proteins in hepatoma cells [69]. Similarly, crizotinib markedly elevates ROS levels to promote autophagy activation and pyroptosis, mediating hepatotoxicity during the therapy process [73]. In addition, autophagy caused by TKIs can also induce ferroptosis of killer cells. For example, neratinib increases ROS and Fe^2+^ activities and downregulates GPX4, leading to ferroptosis of AML cells [31, 74].

Unlike toxic autophagy, ROS-induced protective autophagy mediates the insensitivity of cancer cells to TKIs. Afatinib inhibits mTOR through the ROS/DNA damage responses 1/tuberous sclerosis 1 (ROS/REDD1/TSC1) axis, stimulates protective autophagy in HNSCC cells, and diminishes their susceptibility to cell death [67]. In lung cancer cells, the accumulation of ROS caused by afatinib treatment led to the downregulation of AKT/mTOR signal transduction and produces protective autophagy; however, the combination of autophagy inhibitors enhanced the therapeutic efficacy of afatinib [29]. Vandetanib, a multitarget tyrosine kinase inhibitor, induces protective autophagy and leads to chemical resistance by increasing the levels of ROS in NSCLC cells [75]. In HNSCC, erlotinib upregulated the expression of NOX4 and caused ROS production, which not only induces toxicity but also a degree of protective autophagy [17]. Therefore, the application of autophagy inhibitors has become an effective method of blocking protective autophagy and improving TKI responsiveness. The combination of autophagy inhibitors and TKIs can drive the differentiation of primitive cells and sensitize imatinib-resistant leukemia stem cells [76]. Additionally, pairing an autophagy inhibitor with erlotinib improves the efficacy of HNSCC therapies [17].

In summary, although the role of autophagy in TKI treatment is very complex, promoting ROS accumulation and causing toxic autophagy or using autophagy inhibitors to block ROS-induced protective autophagy effectively improves the sensitivity of cancer cells to TKIs. The specific application of autophagy in tumor cells may be related to factors such as cell type and tumor stage, and further exploration is needed.

#### NRF2-KEAP1 pathway

The transcription factor nuclear factor erythroid 2 related factor 2 (NRF2) is considered one of the main mediators of the cellular antioxidant response. The main function of NRF2 is activating the cellular antioxidant response by inducing the transcription of target genes [77–79]. Kelch ECH-associated protein 1 (KEAP1) serves as a substrate adaptor protein for a cullin 3-containing E3 ubiquitin ligase that binds to NRF2 as a dimer, mediating the ubiquitination degradation of NRF2 [77, 80]. Osimertinib exposure leads to an upregulation of suppressor of cytokine signaling 3 (SOCS3), which competes with NRF2 for KEAP1 binding, diminishing NRF2 degradation, activating antioxidant pathways, and contributing to osimertinib resistance in lung cancer [19]. Lapatinib treatment activates the accumulation of the NRF2- KEAP1 pathway in hepatoma cells via ROS [30]. Similarly, activation of the NRF2- KEAP1 pathway is also observed in TKI treatments of lung cancer, breast cancer and kidney cancer, and is related to the emergence of drug resistance [37, 81, 82]. Berberine counteracts lapatinib resistance in breast cancer by reducing c-Myc levels and disrupting NRF2 stability [82]. Bexarotene, an NRF2 inhibitor, enhances the effectiveness of HER1 blockade when used with HER1 inhibitors such as lapatinib or erlotinib [83]. Silencing KEAP1 reverses the lethality of axitinib in RCC, while silencing NRF2 increases the sensitivity of RCC to axitinib [37]. The covalent JNK inhibitor JNK-IN-8 overcomes NRF2 activation caused by lapatinib treatment by inhibiting the JNK pathway, increasing ROS levels and promoting triple negative breast cancer cell apoptosis [84]. The downstream target gene of NRF2 was also found to be involved in TKI resistance; for instance, glutaredoxin was found to be upregulated in gefitinib-resistant cells [85], and heme oxygenase-1 (HO-1) was upregulated in osimertinib-resistant cells [86]. The antimalarial drug dihydroartemisinin (DHA) reduces the expression of HO-1 in osimertinib-resistant NSCLC cells, inhibiting cell proliferation and cooperating with osimertinib to improve ROS levels and reverse the resistance of NSCLC to osimertinib [86]. Additionally, siramesine and lapatinib in combination synergistically induce ferroptosis through HO-1 degradation, increased ROS, and lipid peroxidation [87].

**Fig. 3:**
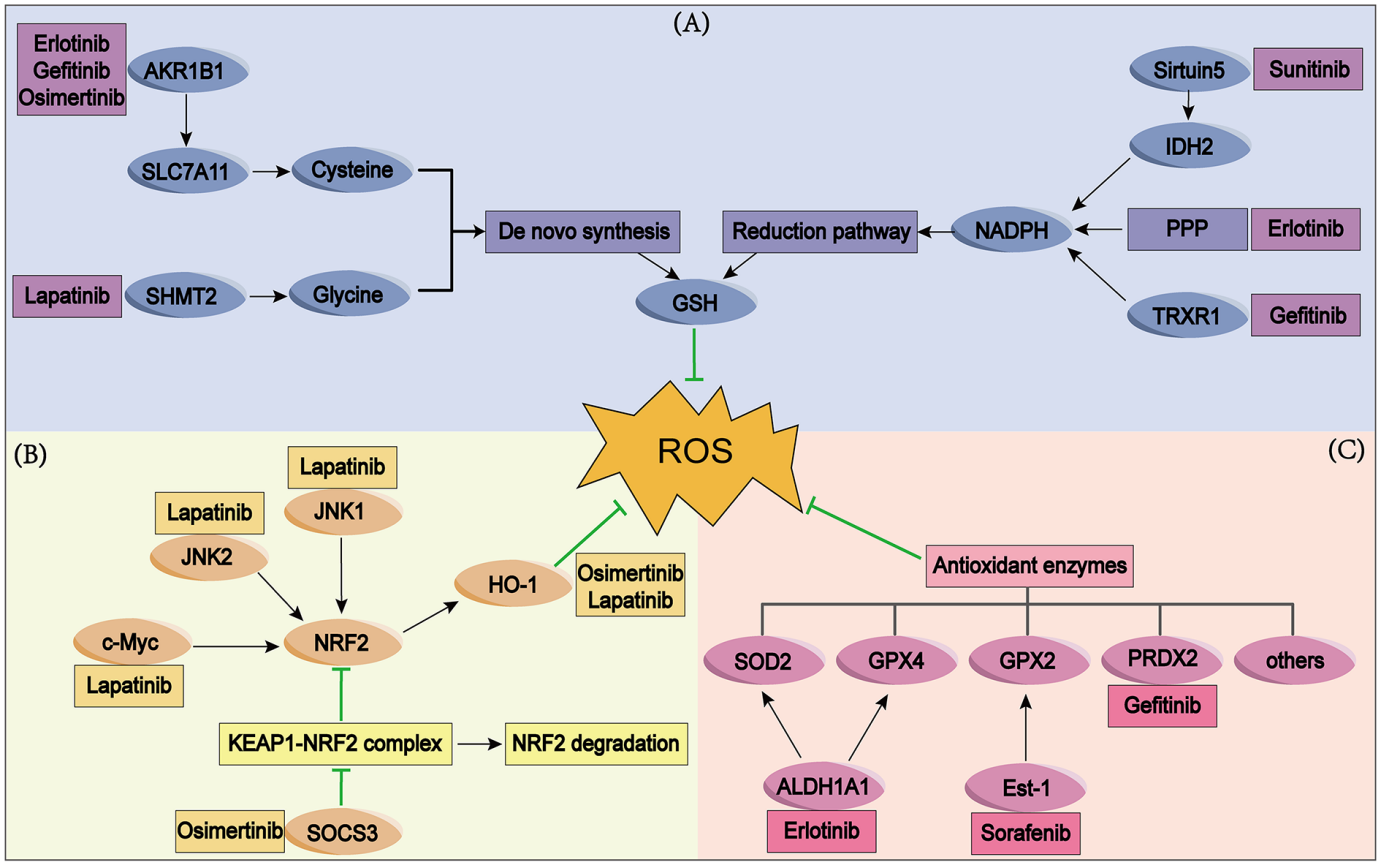
Activated antioxidant pathways in TKI resistant cancer cells. (**A**) There are two primary pathways for GSH production: de novo synthesis and GSSG reduction. In TKI resistant cancer cells, AKR1B1 promotes cysteine transport, SHMT2 enhances glycine production, and both cysteine and glycine serve as substrates for stimulating de novo synthesis of GSH. In addition, Sirtuin5, TRXR1 and PPP contributes to the reduction pathway of GSH by increasing NADPH levels. (**B**) JNK1, JNK2 and c-Myc upregulate NRF2. SOCS3 upregulates NRF2 by inhibiting the NRF2-KEAP1 combination. Upregulated NRF2 then exerts antioxidant effects through downstream molecules like HO-1. (**C**) Antioxidant enzymes increase in TKI resistant cells and subsequently suppress intracellular ROS. The boxes display the corresponding TKIs

The accumulation of ROS generated during TKI treatment induces activation of the intracellular NRF2-KEAP1 pathway. NRF2 regulates the expression of downstream target genes through transcriptional regulation to produce antioxidant effects that counteract ROS’s harmful impact on cells [88, 89], which is one of the mechanisms of TKI resistance (Fig. 3). Therefore, targeting the NRF2-KEAP1 pathway in drug-resistant cancer cells has emerged as a viable strategy to overcome TKI resistance.

#### Glutathione metabolism

Glutathione (GSH) is the most abundant antioxidant in the body. It interacts with ROS to form disulfide-oxidized (GSSG) forms under the catalysis of GPXs, thereby clearing excess ROS in cells and shielding them from oxidative stress [90]. There are two main ways to generate GSH: first one is the two-step ATP-dependent enzymatic reaction catalyzed by glutamate cysteine ligase and GSH synthetase with cysteine and glutamic acid as substrates; second, GSSG is reduced to GSH with the assistance of GSH reductase and nicotinamide adenine dinucleotide phosphate (NADPH) [91, 92]. Changes in substrates, cofactors, and enzymes during the synthesis of GSH impact intracellular GSH levels and modify cellular resilience to ROS (Fig. 3). Studies have shown that glutathione metabolism is involved in TKI resistance. Aldo-keto reductase family 1 member B1 (AKR1B1) is upregulated in gefitinib-, erlotinib-, and osimertinib-resistant lung cancer cells and accelerates the de novo synthesis of glutathione by promoting the cystine transporter solute carrier family 7 member 11 (SLC7A11) expression, thereby reducing treatment-induced stress, such as ROS accumulation, ultimately leading to resistance [93]. The activated metabolism of the pentose phosphate pathway (PPP) generates NADPH, increases the intracellular GSH/GSSG ratio and protects cells from ROS-induced damage, contributing to erlotinib resistance in pancreatic cancer [94] and imatinib resistance in gastrointestinal stromal tumors [95]. Sirtuin5 increases the stability of isocitrate dehydrogenase 2 (IDH2) at the succinylation site K413, resulting in greater NADPH production, which augments the antioxidant defenses of renal cancer cells and their resistance to sunitinib [96]. Likewise, serine hydroxymethyltransferase 2 (SHMT2) activated in lapatinib-resistant breast cancer promotes the synthesis of glycine and increases the synthesis of GSH [97]. In addition, certain drugs can overcome TKI resistance by inhibiting the generation of GSH. For instance, shikonin inhibits thioredoxin reductase 1 (TRXR1), thereby inhibiting the reduction of GSSG to GSH, causing apoptosis of gefitinib-resistant lung cancer cells [98]. Greensporone A produces ROS through depletion of GSH levels, and enhances the activity of imatinib in leukemia cells [99]. Therefore, inhibiting glutathione metabolism in TKI-resistant cells is a good method to induce intracellular ROS accumulation and restore TKI sensitivity.

#### Antioxidant enzymes

Antioxidant enzymes are enzymes that have the ability to convert peroxides into less toxic products, helping cells avoid damage from peroxides, including superoxide dismutase (SOD), glutathione peroxidases (GPX) and peroxiredoxin (PRDX) [100, 101]. In erlotinib-resistant lung cancer cells, the upregulated expression of SOD2 and GPX4 induced by aldehyde dehydrogenase 1A1 (ALDH1A1) reduces the ROS-RCS levels caused by erlotinib and endows TKI resistance [102]. PRDX2 is overexpressed and highly demethylated in gefitinib-resistant A549 cells, causing a decrease in ROS and participating in JNK phosphorylation and the apoptosis signaling pathway, thus playing an important role in cancer cell survival [103]. In HCC, the rise of transcription factor E26 transformation-specific-1 (Ets-1) leads to the expression of GPX2, causing mitochondrial damage and a significant reduction in mitochondrial ROS production, resulting in sorafenib resistance [104]. GPX and catalase activity seem elevated in imatinib-resistant CML cells compared to sensitive ones [105]. Increasing antioxidant enzymes in drug-resistant cells participates in the occurrence of TKI resistance by reducing ROS damage (Fig. 3), so inhibiting antioxidant enzyme activity in drug-resistant cells may be a potential method to overcome TKI resistance.

### Increasing ROS production to overcome TKI resistance

NADPH oxidases are important enzymes mediating the production of hydrogen peroxide and peroxides within the body [106]. The levels of cellular ROS are affected by their expression and activity. In previous explanations, it has been shown that the NOX family plays a role in the TKI-induced cell killing process [14, 16, 42, 47]. Additionally, activated NOX family enzymes lead to the death of drug-resistant cells. Combining erlotinib with ampelopsin induces caspase-dependent apoptosis through the NOX2-ROS-Bim pathway, overcoming resistance to erlotinib in NSCLC cells [107]. 6-Shogaol stimulates ROS production via NOX4 in ovarian cancer, leading to ER stress, eIF2α phosphorylation, and upregulation of activating transcription factor 4 and C/EBP homologous protein (CHOP), which induces apoptosis and overcomes gefitinib resistance [108]. In EGFR^T790M^ TKI-resistant NSCLC cells, sanguinarine activates NOX3, leading to the accumulation of ROS, resulting in NADPH depletion, causing methionine reductase A (MsrA) to destroy its protein reduction protection against methionine 790 of EGFR, causing EGFR peroxidation and degradation, and inducing cancer cell apoptosis [109]. Therefore, activating the NOX family to increase ROS production in drug-resistant cells is also a choice to overcome TKI resistance.

### Providing exogenous ROS stimulation to overcome TKI resistance

In addition to influencing the production and clearance of endogenous ROS, the use of exogenous ROS heightens the sensitivity of cancer cells to TKIs. Photodynamic therapy (PDT) serves as a notable method for such intervention. PDT works by accumulating photosensitizers in tumor cells that release energy when exposed to specific wavelengths of light, leading to the production of ROS and subsequent cytotoxicity [110, 111]. This process resembles how TKIs promote cell death through ROS induction. Hence, combining TKIs with PDT effectively enhances ROS accumulation in tumors, strengthens cytotoxic effects, and improves tumor cell sensitivity to TKIs. This approach has shown promise in various cancers including lung [112, 113], prostate [114], renal cell [115], colorectal adenocarcinoma [116], and liver cancer [117]. Moreover, PDT has been found to complement axitinib in tumor suppression by damaging tumor blood vessels [118], and to augment the immune response when used with dasatinib [119].

### Other methods

Besides the primary anti-ROS or pro-ROS production pathways that target TKI resistance, this section will also mention some potential resistance mechanisms related to intracellular ROS. Although the relationship between these mechanisms and ROS is not very clear, it is evident they play a part in the ROS-related TKI resistance process, like when combining HDAC inhibitors with TKIs.

Histone acetylase causes cancer progression by acetylating histones and inhibiting gene expression, while histone acetylation inhibitors (HDACis) inhibit tumor progression by blocking HDAC, regulating cell cycle arrest, chemical sensitization, apoptosis, and upregulation of tumor suppressors [120, 121]. Treating tumors with HDACis stimulates the generation of ROS, triggers the intrinsic pathway of apoptosis, and is linked to cell death [122, 123]. For instance, the HDACi vorinostat enhances the therapeutic effect of gefitinib or erlotinib, resulting in strong synergistic anti-proliferation and pro-apoptotic effects, overcoming EGFR-TKI resistance. This synergy is associated with the accumulation of reactive oxygen species and increased DNA damage [124, 125]. Overexpression of HO-1 activates HDACs, which diminishes ROS levels and causes imatinib resistance in CML cells [126]. Combining HDACis with imatinib in resistant leukemia cells greatly increases mitochondrial damage and cell death, potentially because it prevents the acetylated heat shock protein 90 from binding to BCR-ABL, aiding in the proteasomal degradation of BCR-ABL protein [127, 128]. Additionally, when HDACis are used alongside other drugs, they contribute to the destruction of imatinib-resistant BCR-ABL positive cells, as seen in combinations with polo-like kinase 1 inhibitors [129], the dual BCR-ABL/Aurora kinase inhibitor KW-2449 [130], and the proteasome inhibitor bortezomib [131]. Although the mechanism of ROS production mediated by HDACis is unclear, the potential of HDACis combined with TKI in overcoming drug resistance cannot be denied, and is worthy of further mechanistic exploration.

Combination therapy can effectively overcome TKI resistance [132], and therefore, identifying suitable treatment pairings is a viable strategy to overcome TKI resistance. Similar to the drug combinations of HDACis with TKIs, there are other therapeutic pairings which enhance ROS accumulation in cancer cells, thereby increasing the efficacy of TKIs. In Table 3, we have listed several such therapeutic combinations.

**Table 3:** Combination therapeutic options with TKIs to enhance ROS accumulation in cancer cells

Addition therapy	Combined TKI	Cancer	References
HADCi	Gefitinib	NSCLC	[125]
	Neratinib	Uveal melanoma	[133]
	Lenvatinib	Liver cancer	[69]
	Dasatinib	CML	[127]
Chloroquine (autophagy inhibitor)	Afatinib	Lung adenocarcinoma	[29]
	Erlotinib	HNSCC	[17]
	Imatinib	CML	[76]
Photodynamic therapy	Lenvatinib	Liver cancer	[117]
	Dasatinib	Acute lymphoblastic leukemia	[119]
Obatoclax (BCL-2 family antagonist)	Lapatinib	Breast cancer	[71, 72]
Siramesine (Lysosomotropic agent)	Lapatinib	Advanced prostate cancer	[134]
	Lapatinib	Breast cancer	[135]
	Lapatinib	Glioblastoma, lung adenocarcinoma	[87]
Retinoid (NRF2 inhibitor)	Lapatinib, erlotinib	Ovarian Cancer	[83]
JNK-IN-8 (JNK inhibitor)	Lapatinib	Breast cancer	[84]
Auranofin (TXNRD1 inhibitor)	Erlotinib	NSCLC	[136]
Topotecan (topoisomerase inhibitor)	Crizotinib	NSCLC	[137]
Cladribine (adenosine deaminase inhibitor)	Gefitinib, dasatinib	Breast cancer	[138]
Ezatiostat (GSTP1 inhibitor)	Crizotinib	Lung adenocarcinoma	[139]

Some studies have found other TKI resistance mechanisms associated with ROS. Treatment of breast cancer cells with the lysosome-disrupting agent siramesine alongside lapatinib elevates FeCl3 levels, diminishes the iron transport protein ferroportin 1, raises cytosolic ROS, and induces ferroptosis [135]. In osimertinib-induced drug-resistant persistent cells, miR-21-5p is upregulated, and adenylosuccinate lyase, an essential enzyme in the de novo purine biosynthesis pathway, is inhibited. The inhibition of ADSL prevents the generation of acadesine, leading to low ROS levels in drug-resistant persistent cells [140]. The overexpression of NADH dehydrogenase (ubiquinone) 1 alpha subcomplex 4-like 2 (NDUFA4L2) promotes mitochondrial relocalization of HER2 and inhibits the production of ROS, thus making HER2-positive breast cancer cells more resistant to herceptin treatment [141]. Elevated ROS activity in lenvatinib-resistant cells is involved in inducing EGFR activation, leading to drug resistance in HCC cells [142]. In erdafitinib-resistant bladder cancer cells, high levels of prolyl 4-hydroxylase subunit alpha 2 (P4HA2) stabilize hypoxia inducible factor 1α (HIF-1α), which activates downstream target genes and lowers ROS levels in bladder cancer [53]. In contrast, sustained activation of signal transducer and activator of transcription 5 causes an increase in ROS accumulation and chronic oxidative damage to DNA, leading to the accumulation of mutations and participating in the resistance of CML to imatinib [143]. These complex and diverse mechanisms suggest that ROS play important and variable roles in TKI resistance, requiring further study based on more specific tumor types and TKI types.

In addition to these studies on specific mechanisms, certain studies have found that the combination of some compounds and TKIs may eradicate drug-resistant cancer cells through causing ROS accumulation-related effects such as ES stress, mitochondrial damage, and apoptosis (Table 4). By combining these natural extracts or drugs that have been clinically applied with TKIs, better safety can be achieved while enhancing TKI efficacy, which is beneficial for clinical conversion.

**Table 4:** Compounds overcoming TKI resistance through ROS-related mechanisms in cancer

Compound name	TKI	Cancer	Regulated targets	Cellular effects	References
Celastrol	Afatinib	NSCLC	Upregulated UBE1 and XBP1	Enhanced cell paraptosis	[144]
Shikonin	Erlotinib Gefitinib	NSCLC	Unspecified	Enhanced ER stress and cell apoptosis	[145]
Ampelopsin	Erlotinib	NSCLC	Upregulated NOX2	Enhanced cell apoptosis	[107]
Plumbagin	Gefitinib	NSCLC	Unspecified	Enhanced DNA damage, cell cycle arrest and cell apoptosis	[146, 147]
Benzyl isothiocyanate	Gefitinib	NSCLC	Repressed Akt Phosphorylation and enhanced MAPK Phosphorylation	Enhanced cell apoptosis and inhibited cell growth	[148]
6-Shogaol	Gefitinib	ovarian cancer	Upregulated NOX4	Enhanced ER stress and cell apoptosis	[108]
Shikonin	Gefitinib	NSCLC	Inhibited TRXR1	Enhanced cell apoptosis	[98]
Dihydroartemisinin	Gefitinib	NSCLC	Unspecified	Enhanced cell apoptosis and ferroptosis	[149]
Dihydroartemisinin	Osimertinib	NSCLC	Inhibited HO-1	Enhanced cell apoptosis	[86]
Bazedoxifene	Osimertinib	NSCLC	Inhibited SOCS3	Enhanced cell apoptosis	[19]
β‑Escin	Trastuzumab	breast cancer	Increased active p18Bax fragmentation	Enhanced cell apoptosis	[150]
Topotecan	Crizotinib	NSCLC	Inactivated EGFR downstream signaling pathways	Enhanced cell apoptosis	[137]
AKI603	Imatinib	CML	Unspecified	Enhanced cell senescence	[151]
PEITC	Imatinib	CML	Enhanced degradation of BCR-ABL	Enhanced cell apoptosis	[152]
Sulforaphane	Imatinib	CML	Inhibited GSTP1	Enhanced cell apoptosis	[153]

Beyond the scope of the first three generations of TKI drugs discussed in this paper, the emerging fourth-generation TKI drugs also warrant attention. These fourth-generation TKIs are primarily designed to overcome mutations that have arisen in the targets of first three generations of TKI therapies [154]. Notable examples include drugs developed to target the third-generation EGFR-TKI C797S mutation, such as BLU-945 and EAI045 [155, 156]. Additionally, compounds such as TPX-0131 and NVL-655 are being designed to address dual-mutation positive ALK subtypes that previous ALK-TKIs have been unable to effectively target [157]. Nonetheless, these drugs are currently in the clinical research phase, and their anti-tumor efficacy awaits further validation through additional clinical data. Furthermore, the role of ROS in the tumoricidal mechanisms induced by these novel TKIs and its potential impact on drug sensitivity require further investigation upon the market availability of these drugs and subsequent in-depth studies.

## Conclusion and future perspectives

TKIs are pivotal in modern cancer therapy, as they elevate intracellular ROS, which damages DNA, proteins, and organelles, ultimately causing cancer cell death. This phenomenon is commonly observed during TKI action against cancer cells. Cancer cells often respond to oxidative stress by activating antioxidant pathways for self-protection, and these pathways can lead to TKI resistance. By inhibiting these pathways, the response of drug-resistant cancer cells to TKIs may improve. Additionally, stimulating ROS generation within drug-resistant cells or providing exogenous ROS stimulation is also an effective means to overcome TKI resistance. Although it is evident that ROS plays a vital role in TKI resistance, the specific mechanisms of ROS-related cell death or resistance are not fully understood due to ROS complexity and variations in tumor origin, microenvironment, and stage. Hence, precise and in-depth research is needed. In summary, promoting ROS accumulation to overcome TKI resistance is a universal and effective method, and deeper exploration of the mechanisms can provide opportunities to identify critical therapeutic targets.

## Data Availability

Not applicable.

## Abbreviations

AKR1B1: Aldo-keto reductase family 1 member B1
AKT: Protein kinase B
ALDH1A1: Aldehyde dehydrogenase 1A1
ALK: Anaplastic lymphoma kinase
AML: Acute myeloid leukemia
AMPK: Adenosine monophosphate-activated protein kinase
Ask1: Apoptosis signal-regulating kinase 1
ATM: Ataxia telangiectasia mutated
CHOP: C/EBP homologous protein
CML: Chronic myeloid leukemia
DHA: Dihydroartemisinin
EGFR: Epidermal growth factor receptor
eIF2α: Elongation initiation factor 2α
EMT: Epithelial-mesenchymal transition
ER: Endoplasmic reticulum
Ets-1: E26 transformation–specific-1
FGFR: Fibroblast growth factor receptor
FLIP: FLICE-like inhibitory protein
GPX: Glutathione peroxidase
GSH: Glutathione
GSK3β: Glycogen synthase kinase 3β
GSSG: Disulfide-oxidized
GSTP1: Glutathione S-transferase pi
H_2_O_2_: Hydrogen peroxide
HCC: Hepatocellular carcinoma
HDAC: Histone deacetylase
HDACi: Histone acetylation inhibitor
HER2: Human epidermal growth factor receptor 2
HIF-1α: Hypoxia inducible factor 1α
HNSCC: Head and neck cancer
HO-1: Heme oxygenase-1
IDH2: Isocitrate dehydrogenase 2
JNK: C-Jun N-Terminal kinase
KEAP1: Kelch ECH-associated protein 1
MsrA: Methionine reductase A
mTOR: Mammalian target of rapamycin
NADPH: Nicotinamide adenine dinucleotide phosphate
NDUFA4L2: NADH dehydrogenase (ubiquinone) 1 alpha subcomplex 4-like 2
NOX: NADPH oxidase
NRF2: Nuclear factor erythroid 2 related factor 2
NSCLC: Non-small cell lung cancer
O_2_: Superoxide
OH^•^: Hydroxyl radical
P4HA2: Prolyl 4-hydroxylase subunit alpha 2
PARP: Poly (ADP-ribose) polymerase
PDGFR: Platelet-derived growth factor receptor
PDT: Photodynamic therapy
PPP: Pentose phosphate pathway
PRDX: Peroxiredoxin
RCC: Renal cell carcinoma
REDD1: DNA damage responses 1
ROS: Reactive oxygen species
SHMT2: Serine hydroxymethyltransferase 2
SLC7A11: Solute carrier family 7 member 11
SOCS3: Suppressor of cytokine signaling 3
SOD: Superoxide dismutase
TIE-2: TEK receptor tyrosine kinase
TKI: Tyrosine kinase inhibitor
TRXR1: Thioredoxin reductase 1
TXNRD1: Thioredoxin reductase 1
UBE1: Ubiquitin-activating enzyme 1
TSC1: Tuberous sclerosis 1
VEGFR: Vascular endothelial growth factor receptor
XBP1: X-box binding protein‐1
XIAP: X-linked inhibitor of apoptosis protein
1O2: Singlet oxygen
